# Editorial: Myotonic Dystrophies: Developments in Research From Bench to Bedside

**DOI:** 10.3389/fneur.2020.594836

**Published:** 2020-09-30

**Authors:** Gabriella Silvestri, Federica Montagnese

**Affiliations:** ^1^Department of Neuroscience, Neurology Faculty of Medicine, Catholic University of the Sacred Heart, Milan, Italy; ^2^Neurology Unit, Agostino Gemelli University Polyclinic, Catholic University of the Sacred Heart, Rome, Italy; ^3^Department of Neurology, University Hospital of Munich, München, Germany

**Keywords:** myotonic dystrophy type1, myotonic dystrophy type2, Steinert's disease, myotonic dystrophies, cardiac involvement in DM, outcome measures in DM, CNS involvement in DM, tumor risk in DM

This special issue on myotonic dystrophies (DM) offers a comprehensive overview on the emerging clinical Research Topics in this field.

Two reviews are focused on two major extramuscular manifestations in DM, giving important suggestions regarding the related clinical management, and also illustrating pathophysiological data obtained by the use innovative diagnostic techniques, being potentially applicable in view of future therapeutic perspectives: in particular Nieuwenhuis et al. revised the current knowledge regarding the etiology and the role of the insulin resistance, discussing the consequences of the altered insulin signaling in the myotonic dystrophies. This topic is actually relevant not only for glucose metabolism, but also for the other insulin dependent muscle and brain signaling pathways, as it could play a role in the pathogenesis of related clinical features in myotonic dystrophies, and thus it justifies further basic scientific and clinical, therapeutically oriented research that may modify DM1 symptom outcomes, as in the case of type II diabetes medication lowering insulin resistance metformin.

Simoncini et al. gave a comprehensive review of the most significant research concerning the central nervous system involvement in DM1 and DM2, their clinical and neuroimaging correlates and the ongoing search of reliable outcome CNS measures suitable for any future therapeutic clinical trial.

Regarding feasible outcome measures to be used “at the bedside,” of DM patients the research work by Montagnese et al. fulfills a gap regarding the lack of disease-specific, validated, motor outcome measures (OM) and patients' reported outcomes (PRO) in DM2; they tested 66 DM2 patients at a baseline and after 1 year follow-up by several OMS and PRO already applied in other neuromuscular disorders. Myotonia behavior scale (MBS), manual muscle testing (MMT), hand held dynamometry (HHD), and 6MWT resulted meaningful motor outcome measures in DM2 also in view of future therapeutic trials.

Given the slow progression of the myotonic dystrophies there is need of assessing the potential role of novel and more sophisticated diagnostic approaches to detect and monitor more subtle tissues changes in a time span suitable for future therapeutic trials. In this regard three contributors have been focused on the clinical assessment of CNS and cardiac involvement in myotonic dystrophies: Ates et al. describe the results of a prospective, explorative study to assess iron accumulation in the deep gray matter in relation to progressive CNS involvement in 16 DM1 and 12 DM2 patients, by the use of quantitative mapping of the magnetic susceptibility and the effective transverse relaxation rate (R2^*^) in distinct brain regions; susceptibility, R2^*^ and volumes, determined for 11 DGM structures were compared between patients and controls. This study shows the presence of iron accumulation, likely related to neurodegeneration, in some deep gray matter nuclei of DM patients, being more widespread in DM1, and correlating either with age (greater in adult vs. childhood DM1) and with some clinical features and with specific cognitive deficits in both forms.

As neuronal iron accumulation can be found in aged brains by MRI, the results of this study further support the definition of myotonic dystrophies as “progeroid” diseases, even if it's true that an aberrant increase of fetal mRNA isoforms in DM patients' tissues represent the characteristic molecular hallmark both in DM1 and DM2. ON the other hand, the results presented in their work by Serra et al. would reinforce the idea of DM1 as being also a neurodevelopmental disorder in which brain abnormalities might account for their social cognition deficits. These authors investigated the cortical thickness and its potential association with social cognition deficits in 30 DM1 patients vs. 26 controls using a 3T MRI scan and a specific battery of tests the Social Cognition Battery. DM1 patients showed low performances in several subtests, and a reduced thickness in the right premotor cortex, angular gyrus, precuneus, and inferior parietal lobule, findings that were also significantly associated with their individual genetic load.

Regarding cardiac involvement, in their brief research article, Alì et al. evaluated myocardial strain and extracellular volume in DM1 patients as potential imaging biomarkers of subclinical cardiac pathology by a retrospective study on 9 DM1 patients without apparent cardiac disease compared with age- and sex-matched healthy controls. DM1 patients showed a significant increase in cardiac extracellular volume and decrease in strain, pointing out to the sensitivity of cardiac magnetic resonance in identifying early signs of cardiac involvement in DM1 and thus its potential usefulness to assess the progression of myocardial pathology longitudinally.

Gastrointestinal symptoms often affect the quality of life in DM1 patients; Perna et al. assessed prevalence, spectrum and potential determinants of the involvement of the gastrointestinal system in DM1 by studying a cohort of 61 DM1 patients by an extensive diagnostic protocol including in particular administration of a validated patient-reported questionnaire about GI symptoms (GSRS), liver US scan and intestinal permeability assay. Besides confirming a high frequency of various gastrointestinal symptoms, this study reveals the presence of an altered intestinal permeability by a specific radionuclide-based assay in more than 90% of DM1 patients studied: this finding could support the potential contribution of an altered microbiota in the pathogenesis of insulin resistance in DM1. These authors have also documented a gender-related prevalence and severity of specific gastrointestinal manifestations, issue to consider when analyzing results of future therapeutic trials on specific DM1 manifestations.

Finally, the pathogenesis of an increased tumor risk in DM patients, recently documented by several studies is still unknown; of note Higgs et al., leaders in this research field, have characterized a wide range of reproductive risk factors in women with DM in order to assess their role for the increased risk of reproductive organ tumors of DM1 women. Using questionnaires, they collected and analyzed personal history information related to cancer risk factors from 242 DM type 1 (DM1) and 44 DM type 2 (DM2) women enrolled in the UK Registry (*N* = 124) and the US National Registry (*N* = 162). There were no statistically significant differences between DM1 and DM2 regarding menstrual history or fertility-related factors, menopausal hormone therapy (HT) and hysterectomy, after age adjustment. The frequency of self-reported reproductive organ tumors was not significantly different comparing DM1 to DM2, yet women with DM2 appear to have a lower risk of malignant tumors compared to those with DM1. These findings support that the known excesses of ovarian and endometrial cancer reported in women with DM1 might not be attributed to standard cancer-related reproductive risk factors, issue that in any need to be confirmed on larger studies.

In conclusion this Research Topic further has contributed to enrich the current knowledge on the myotonic dystrophies, which represent a complex but fascinating challenge for any clinical and basic researcher working in this field.

## Author Contributions

Both authors contributed to writing of this editorial regarding the Research Topic edited.

### Conflict of Interest

The authors declare that the research was conducted in the absence of any commercial or financial relationships that could be construed as a potential conflict of interest.

